# Signaling function of Na,K-ATPase induced by ouabain against LPS as an inflammation model in hippocampus

**DOI:** 10.1186/s12974-014-0218-z

**Published:** 2014-12-31

**Authors:** Paula Fernanda Kinoshita, Lidia Mitiko Yshii, Andrea Rodrigues Vasconcelos, Ana Maria Marques Orellana, Larissa de Sá Lima, Ana Paula Couto Davel, Luciana Venturini Rossoni, Elisa Mitiko Kawamoto, Cristoforo Scavone

**Affiliations:** Molecular Neuropharmacology Laboratory, Department of Pharmacology, Institute of Biomedical Science, University of São Paulo, 05508-900 São Paulo, Brazil; Department of Physiology and Biophysics, Institute of Biomedical Sciences, University of São Paulo, São Paulo, Brazil

**Keywords:** Ouabain, Na,K-ATPase, TNF-α, NF-κB, Inflammation, LPS

## Abstract

**Background:**

Ouabain (OUA) is a newly recognized hormone that is synthesized in the adrenal cortex and hypothalamus. Low doses of OUA can activate a signaling pathway by interaction with Na,K-ATPase, which is protective against a number of insults. OUA has central and peripheral anti-inflammatory effects. Lipopolysaccharide (LPS), via toll-like receptor 4 activation, is a widely used model to induce systemic inflammation. This study used a low OUA dose to evaluate its effects on inflammation induced by LPS injection in rats.

**Methods:**

Adult male Wistar rats received acute intraperitoneal (ip) OUA (1.8 μg/kg) or saline 20 minutes before LPS (200 μg/kg, ip) or saline injection. Some of the animals had their femoral artery catheterized in order to assess arterial blood pressure values before and after OUA administration. Na,K-ATPase activity, cytokine mRNA levels, apoptosis-related proteins, NF-κB activation brain-derived neurotrophic factor BDNF, corticosterone and TNF-α levels were measured.

**Results:**

OUA pretreatment decreased mRNA levels of the pro-inflammatory cytokines, inducible nitric oxide synthase (iNOS) and IL-1β, which are activated by LPS in the hippocampus, but with no effect on serum measures of these factors. None of these OUA effects were linked to Na,K-ATPase activity. The involvement of the inflammatory transcription factor NF-κB in the OUA effect was indicated by its prevention of LPS-induced nuclear translocation of the NF-κB subunit, RELA (p65), as well as the decreased cytosol levels of the NF-κB inhibitor, IKB, in the hippocampus. OUA pretreatment reversed the LPS-induced glial fibrillary acidic protein (GFAP) activation and associated inflammation in the dentate gyrus. OUA also prevented LPS-induced increases in the hippocampal *Bax/Bcl2* ratio suggesting an anti-apoptotic action in the brain.

**Conclusion:**

Our results suggest that a low dose of OUA has an important anti-inflammatory effect in the rat hippocampus. This effect was associated with decreased GFAP induction by LPS in the dentate gyrus, a brain area linked to adult neurogenesis.

## Introduction

Na,K-ATPase (NKA) is a membrane protein that is essential for the survival of the organism. This enzyme is expressed in all the cells of the human body, having many functions including the maintenance of osmotic balance, cell volume, pH and membrane potential. This occurs by the hydrolysis of an adenosine triphosphate (ATP) molecule leading to the export of three sodium ions and the import of two potassium ions into the cell, which is fundamental for neuronal excitability and cell maintenance [1,2].

NKA is constituted of three subunits: α, β and γ [3], with each subunit having a number of isoforms that provide functional versatility across different cell types, in turn highlighting the different roles and responses produced by NKA activation across cell types [4-8]. However, the γ (gamma) subunit, is not present in all the cells, with the other subunits being required for NKA to be functional [9].

In the adult brain, α_1_ is expressed in all cells, with α_2_ being expressed primarily in astrocytes and α_3_ in neurons [10,11]. Mutations in the α_2_- and α_3_-isoform genes are involved in neurological disorders, such as familial hemiplegic migraine type-2 [12], rapid-onset dystonia [13], alternating hemiplegia of childhood [14] and cerebellar ataxia, areflexia, pes cavus, optic atrophy and sensorineural hearing loss (CAPOS) [15], with genetic variations in NKA also associating with bipolar disorder, suggesting a role for this enzyme in the etiology of this disease [16]. The NKA α-isoform plays a critical role in the modulation of learning and memory, in turn regulating susceptibility to Alzheimer’s disease [17]. Several works show NKA to operate as a receptor and not only as a pump, with a number of intracellular pathway activations driving its effects [18,19].

Ouabain (OUA) is synthesized by the adrenal gland and hypothalamus [20,21] and is likely to have important physiological roles in both the central and peripheral nervous systems [22,23]. OUA binds to NKA in hippocampal astrocytes, activating inositol trisphosphate receptor (InsP3R), which generates calcium oscillations, thereby activating NF-κB [23]. Xie and Askari [24] also showed OUA to act as a signal transducer, by binding to NKA and thereby activating the Ras-Raf-MAPK signaling cascade by the epidermal growth factor receptor (EGFR).

OUA has a dual role, given its dose-dependent response curve effects. A high concentration of OUA can cause cell death, driving neuronal necrosis via NKA inhibition, leading to potassium ion depletion and thereby increasing intracellular sodium and calcium ions [25]. Conversely, low concentrations of OUA (0.01 nM) are protective against kainic acid-induced lesions in the rat striatum, where it reduces apoptosis by increasing Bcl-2 [26]. Similarly, OUA affords protection in rat kidney primary cultures against Shiga toxin [27]. As such, OUA can afford protection both peripherally and centrally.

NF-κB is a nuclear transcription factor, which is commonly induced following danger or inflammatory signaling, including by lipopolysaccharide (LPS) [28]. NF-κB comprises homo- and heterodimers via the combination of the subunits p65 (RELA), p50, p52, c-REL and REL of the REL/NF-κB family of proteins [28]. Different dimer combinations can activate or block distinct gene transcriptions, exemplified by the inhibitory homodimers p50/p50 and p52/p52 [29]. This transcription factor is evolutionarily conserved, driving a wide range of effects that depend on the specific activating stimulus, particular cell type and the cellular phenomenon that it regulates.

When it is not stimulated, NF-κB resides in the cytoplasm in association with the inhibitory protein, IκB (IKB) [30]. When IKB is phosphorylated by IKK (IκB kinase), IKB is degraded by proteasome 26S [31], freeing NF-κB, which can then translocate to the nucleus and bind to the regulatory sequence close to the promotor region, thereby regulating the expression of target genes such as, the pro-apoptotic *Bax*, anti-apoptotic *Bcl2*, inducible nitric oxide synthase (iNOS), several cytokines, manganese-superoxide dismutase (MnSOD) and brain-derived neurotrophic factor (BDNF) [29]. BDNF is an important trophic protein, including in the central nervous system (CNS), where it plays important roles in brain development and plasticity, with its malfunction being involved in many CNS diseases [32]. In the CNS, NF-κB plays a dual role in neurodegenerative diseases, enhancing survival in neurons, whilst driving pathological glial inflammatory processes [33].

LPS is a bacterial Gram-negative membrane element which is released in a free form or in aggregates. LPS is classically utilized as a model of inflammation and sepsis, with effects via toll-like receptor 4 (TLR4) [34,35]. Peripheral LPS administration induces NF-κB activation in different brain areas [36], as well as *de novo* synthesis of *Iκb* mRNA (an NF-κB activation index), in the brain parenchyma [37]. Peripheral LPS can, therefore, activate pro-inflammatory CNS genes [38]. The present work aims to evaluate the alterations induced by a low dose of OUA in the rat hippocampus, in an inflammation model induced by intraperitoneal (ip) LPS injection.

## Methods

### Animal and tissue preparation

Three-month old male Wistar rats (Biomedical Sciences Institute, University of São Paulo) were kept under 12 hour light/dark cycle (lights on at 7:00 am) and allowed free access to food and water. Animals were treated with OUA (1.8 μg/kg, ip) or saline 20 minutes before the injection LPS (200 μg/kg, ip) or saline and euthanized 2 hours after the administration (between 9:00 and 11:00 am) following procedures approved by the Biomedical College of Animal Experimentation (COBEA). All procedures were also approved by the Ethical Committee for Animal Research (CEEA) of the Biomedical Sciences Institute of the University of São Paulo. The brain was immediately removed and immersed in cold PBS. Each hippocampus was rapidly dissected, quickly immersed in liquid nitrogen, and stored at −80°C for later use.

### Hemodynamic parameters

Male Wistar rats (n = 4) were anesthetized with a ketamine, xylazine and acepromazine mixture (64.9, 3.2 and 0.78 mg/kg). The left femoral artery was cannulated with a polyethylene catheter (PE-10 connected to PE-50 filled with heparinized saline, (BD,New Jersey, NY, USA**)** that was exteriorized in the mid-scapular region. After 24 hours, arterial pressure and heart rate were measured in conscious animals by a pressure transducer (model DT-100, Utah Medical Products, Midvale, UT, USA) and recorded using an interface and software for computer data acquisition (Power Lab 4/25, AD Instruments, Sidney, Australia). Heart rate was determined from the intra-beat intervals. The effects of OUA (1.8 μg/kg, ip) on the arterial blood pressure and heart rate, was evaluated before and 1 hour after OUA administration in conscious animals. Results are expressed as mean ± SE.

### Chemicals and kits

Routine reagents, OUA and LPS from *Escherichia* c*oli* O111:B4 were purchased from Sigma Chemicals (St. Louis, MO, USA); Bio-Rad protein assay kit was purchased from Bio-Rad (Hercules, CA, USA). Corticosterone, TNF-α and BDNF immunoassay kits were purchased from Enzo Life Sciences, Inc. (Farmingdale, NY, USA), eBioscience (San Diego, CA, USA) and Promega (Fitchburg, WI, USA), respectively. The kits were utilized according to manufacturer instructions. All solutions were prepared immediately before use.

### Semiquantitative RT-PCR determination of inducible nitric oxide synthase (*iNos*), *Il*-*1β*, *Bax* and *Bcl2* mRNA levels

The effect of OUA on LPS-modulated gene expression in the hippocampus of rats was measured. Total RNA was isolated with Trizol reagent (Invitrogen, Carlsbad, CA, USA) from the hippocampus according to the manufacturer's instructions. Semiquantitative reverse transcription-PCR (RT-PCR) amplification was performed using the ThermoScript RT kit (Invitrogen, Carlsbad, CA, USA) according to the manufacturer's instructions. The primer sequences were: *iNos* (GenBank access number 012611.3, 651 bp) 5′-GTGCTAATGCGGAAGGTCATA-3′ (sense) and 5′CCAAATGTGCTTGTCACCACA-3′ (antisense); *Bax* (GenBank access number 017059.1, 260 bp), 5^′-^TGAACTGGACAACAACATGGAGC-3′ (sense) and 5′-GGTCTTGGATCCAGACAAACAGC-3′ (antisense); *Bcl2* (GenBank access number 016993.1, 271 bp), 5-′GGAGGATTGTGGCCTTCTTTGAG-3′ (sense) and 5′-TATGCACCCAGAGTGATGCAGGC-3′(antisense); *Il*-*1β* (GenBank access number 031512.2, 1,339 bp), 5′-ATGCTCAGCAGTCAAGTGCC-3′ (sense) and 5′-AGCCTT CCTTCGTGTAACCC-3′ (antisense).

The PCR conditions consisted of 5 minutes at 94°C, 33 cycles of 94°C for 45 seconds, 63 °C for 45 seconds, and 72°C for 1 minute and 30 seconds and a final extension at 72°C for 10 minutes. Glyceraldehyde-3-phosphate dehydrogenase (*Gapdh*; GenBank access number 017008.3, 264 bp) was also amplified as an internal PCR control using the following primers: 5′-CGGGAAGCTTGTGATCAATGG-3′ (sense) and 5′-GGCAGTGATGCCATGGACTG-3′ (antisense). In this case, the temperature cycling conditions were as follows: 5 minutes at 94°C, 20 cycles of 94°C for 45 seconds, 63°C for 45 seconds, and 72°C for 1 minute and 30 seconds and a final extension at 72°C for 10 minutes. Gel electrophoresis of the PCR product was performed using an ethidium bromide-containing agarose gel (2%), and resulting bands were visualized under UV light.

The photographs were captured by photo documentation system DP-001-FDC (VilberLourmat, Torcy, France), and the optical density of the bands was determined using NIH ImageJ software (http://rsb.info.nih.gov/ij). Results were expressed in relation to the intensity of *Gapdh* mRNA levels.

### Protein extraction

Nuclear extract of each hippocampus was prepared as previously described [39]. Briefly, hippocampal structures were homogenized using a Dounce homogenizer in cold PBS supplemented with 0.5 mM dithiothreitol (DTT), 0.5 mM phenylmethylsulfonide fluoride (PMSF), 2 μg/ml leupeptin, 2 μg/ml antipain, and 3 mM sodium orthovanadate and centrifuged at 4°C for 30 seconds at 12,000 g. Supernatant was discarded and pellet was resuspended in lysis buffer (10 mM HEPES, pH 7.9, 1.5 mM MgCl_2_, 10 mM KCl, 0.1 mM ethylenediaminetetraacetic acid (EDTA), 0.5 mM DTT, 0.5 mM PMSF, 2 μg/ml leupeptin, 2 μg/ml antipain, and 3 mM sodium orthovanadate) and incubated on ice for 10 minutes. After addition of NP-40 (10%), samples were vigorously mixed and centrifuged for 30 seconds at 12,000 g. The supernatant was reserved for Western blotting and enzymatic assays (cytosolic fraction), and the pellet was resuspended in lysis buffer (10 mM HEPES, pH 7.9, 1.5 mM MgCl_2_, 10 mM KCl, 0.1 mM EDTA, 0.5 mM DTT, 0.5 mM PMSF, 2 μg/ml leupeptin, 2 μg/ml antipain, and 3 mM sodium orthovanadate) and incubated on ice for 10 minutes. After addition of NP-40 (10%), samples were vigorously mixed and centrifuged for 30 seconds at 12,000 g. Supernatant was discarded, and the pellet was resuspended in extraction buffer (20 mM HEPES, pH 7.9, 25% glycerol, 1.5 mM MgCl_2_, 300 mM NaCl, 0.25 mM EDTA, 0.5 mM DTT, 0.5 mM PMSF, 2 lg/ml leupeptin, 2 lg/ml antipain, and 3 mM sodium orthovanadate), incubated for 20 minutes on ice, and centrifuged for 20 minutes at 12,000 g at 4°C. The resulting supernatants containing nuclear proteins were stored at −80°C and used to test the expression of RELA. Protein concentration was determined using the Bio-Rad protein reagent (Hercules, CA, USA).

### Western blotting

Electrophoresis was performed using 10% polyacrylamide gel and the Bio-Rad mini-Protean III apparatus (Bio-Rad, Hercules, CA, USA). In brief, the proteins present in the hippocampal cytosolic and nuclear fractions were size-separated in 10% SDS-PAGE (90 V). The proteins were blotted onto a nitrocellulose membrane (Bio-Rad, Hercules, CA, USA) and incubated with the specific antibody RELA (p65) (sc-0372; Santa Cruz Biotechnology, Santa Cruz, CA, USA) and IKB (sc-0371; Santa Cruz Biotechnology, Santa Cruz, CA, USA). The Ponceau method to immunoblot was used to ensure equal protein loading [40]. Proteins recognized by antibodies were revealed by an electrochemiluminescence (ECL) technique, following the manufacturer's instructions (Amersham Biosciences, Amersham, UK). To standardize and quantify the immunoblots, we used the photo documentation system DP-001-FDC (VilberLourmat, Torcy, France) and NIH ImageJ software (http://rsb.info.nih.gov/ij). Several exposure times were analyzed to ensure the linearity of the band intensities. β-ACTIN antibody (sc-1616; Santa Cruz Biotechnology, Santa Cruz, CA, USA) was used as an internal experimental control, with results expressed in relation to β-ACTIN intensity.

### Measurement of Na, K-ATPase activity

The NKA activity was determined by assaying Pi released from ATP hydrolysis. This inorganic compound forms a complex with molybdate which can be read spectrophotometrically at 700 nm [41]. For this colorimetric ATP assay, hippocampus supernatant (S1) was centrifuged (12,000 g for 15 minutes at 4°C). The particulate fraction was resuspended in a buffer containing: 0.32 M sucrose, 20 mM HEPES, 1 mM EDTA, 1 mM DTT, and 1 mM PMSF, pH 7.4.

Protein content was determined in particulate samples by colorimetric assay (Bio-Rad, Hercules, CA, USA) [42]. NKA activity was tested by adding 10 μg of the particulate fraction (in 40 μl of buffer) to 360 μl of buffer containing: 3 mM ATP, 120 mM NaCl, 2 mM KCl, 3 mM MgCl_2_, and 30 mM histidine (pH 7.2), with or without OUA (3 μM or 3 mM). After 20 minutes of incubation at 37°C, NKA activity was measured. The reaction was terminated by the addition of a quenching solution (0.6 ml) containing 1 N H_2_SO_4_ and 0.5% ammonium molybdate. Formation of a phosphomolybdate complex was determined spectrophotometrically at 700 nm [43].

The total ATPase, Mg-ATPase, NKA α_1_- and α_2/3_-isoform activities were linearly related up to 20 minutes. In rodents, the NKA α_1_-isoform is 1,000 times less sensitive to the cardiac glycoside than the NKA α_2/3_isoform measured from the difference between OUA-untreated and OUA-treated samples.

The high-affinity α_2/3_-isoform fraction was calculated by subtracting the activity obtained with 3 μM OUA from total-ATPase activity. To determine the low-affinity fraction (α_1_ subunit-associated NKA activity), the values obtained in the presence of 3 μM OUA were subtracted from those obtained in the presence of 3 mM OUA. The NKA activity was expressed as nmol/mg protein x minutes.

### Immunofluorescence in the hippocampus

The brain was fixed with paraformaldehyde and kept in 30% sucrose for 48 hours. Hippocampal coronal sections (30 μm) were cut in the microtome and the tissues collected were kept in 0.1 M PBS. The sections were incubated with 2% normal donkey serum for 2 hours. For immunofluorescence reactions, the sections were incubated overnight with primary antibody (glial fibrillary acidic protein (GFAP), Sigma-Aldrich, St. Louis, MO, USA) and 4',6-diamidino-2-phenylindole; DAPI), followed by 2-hours incubation with secondary antibody (Alexa fluor 488 donkey anti-rabbit for GFAP). Six slices were placed in each slide and mounted with a coverslip. The sections were examined by fluorescence microscopy (Nikon Eclipse 80i with DXM 1200C digital camera; Nikon, Tokyo, Japan).

### Statistical analysis

Results are expressed as mean ± SEM of the indicated number of experiments. Statistical comparisons for OUA-induced changes in mRNA levels, protein expression, ELISA kits and NKA activities were performed by one-way analysis of variance (ANOVA), followed by the Newman-Keuls post-test. All analyses were performed using a Prism 6 software package (GraphPad Software, San Diego, CA, USA). *P*-values < 0.05 were considered to reflect a statistically significant difference.

## Results

### OUA (1.8 μg/kg) does not induce any effect in blood pressure and NKA activity

**Figure 1:**
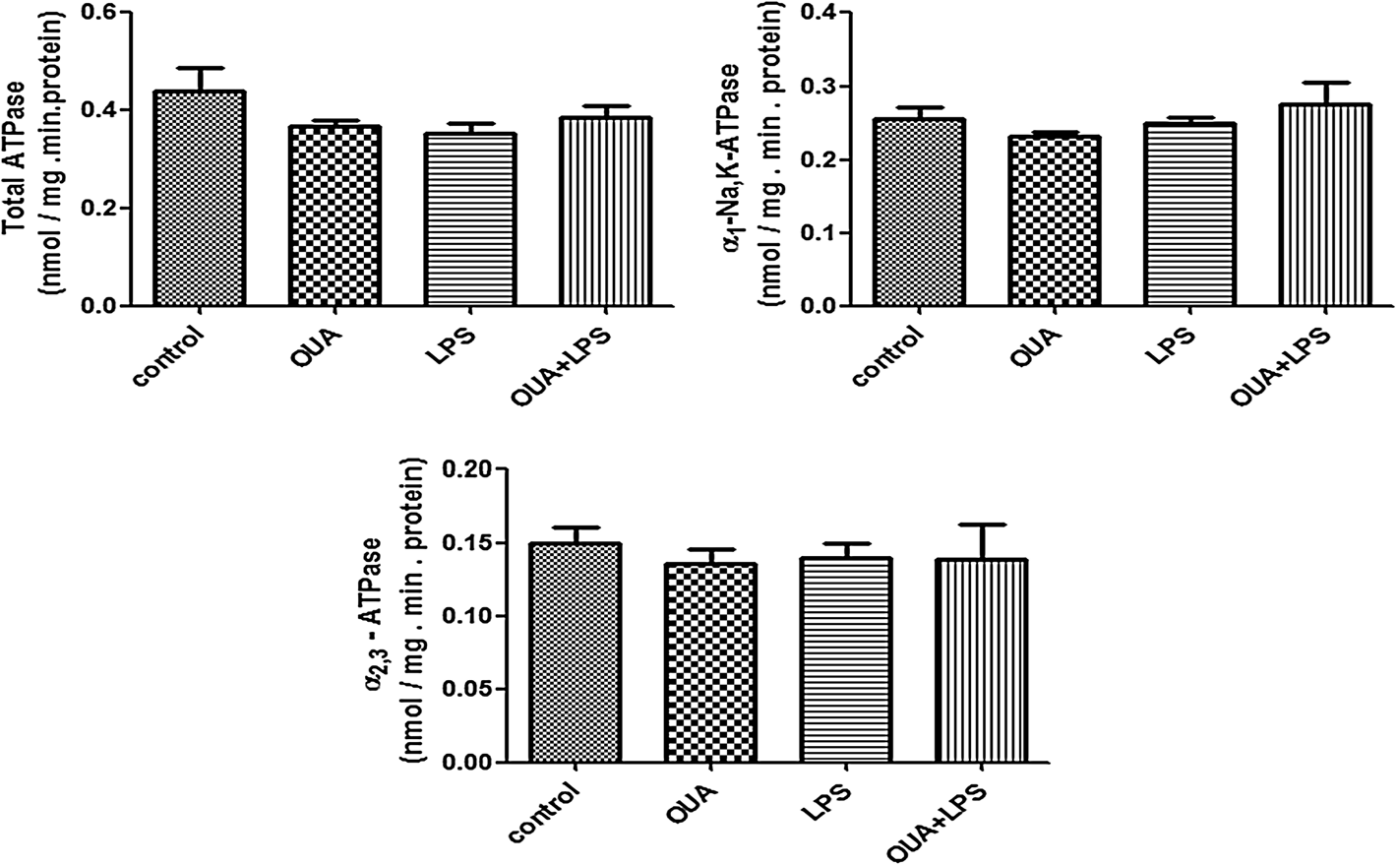
**Pretreatment of ouabain (OUA)** **(1.8 μg/** **kg)** **in lipopolysaccharide (LPS)** **(200 μg/** **kg** **) inflammation model induced no changes on Na**,**K**-**ATPase (NKA) activity.** The total NKA activity was tested by adding 10 μg of the particulate fraction from the rat hippocampus obtained 2 hours after OUA (1.8 μg/kg, ip) or saline pretreatment + LPS (200 μg/kg, ip). α_2/3_-NKA activity was measured by subtracting the activity obtained with 3 μM OUA (it inhibits just these 2 isoforms) from total NKA activity. To determine the α_1_ subunit-associated NKA activity, the total NKA activity was subtracted from the activity obtained with 3 mM OUA (Mg-ATPase). Results are expressed as nmol/mg.min protein (mean + SEM) from five individual experiments. One-way ANOVA followed by Newman-Keuls post-test.

One-hour OUA administration (1.8 μg/kg; ip) did not change mean arterial pressure (Before: 113 ± 3.16 versus After OUA: 112 ± 4.71 mmHg; *P* > 0.05) or heart rate (Before: 323 ± 21.35 versus After OUA: 331 ± 15.46 bpm; *P* > 0.05). In addition, this OUA dose does not change total ATPase, Mg-ATPase, α_1_−NKA and α_2/3−_NKA activities (Figure 1) in the rat hippocampus, suggesting the absence of OUA effects on blood pressure and central NKA activity.

### Pretreatment with OUA reduced *iNos*, *Il*-*1β*, and the *Bax*/*Bcl2* ratio mRNA levels activated by LPS

**Figure 2:**
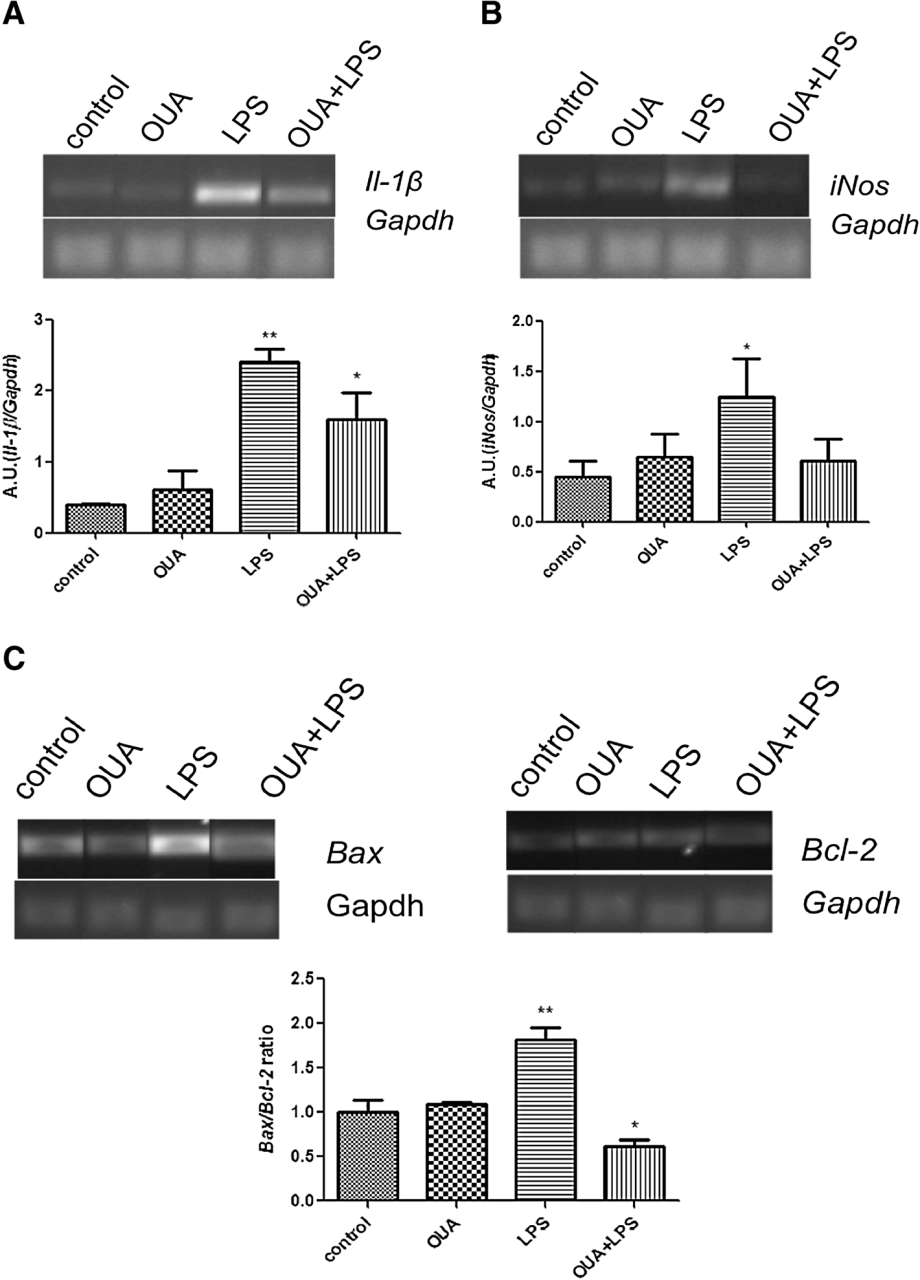
**Effects of pretreatment of ouabain (OUA)** **(1.8 μg/** **kg)** **in the lipopolysaccharide (LPS)** **(200 μg/** **kg)** **inflammation model on the mRNA levels of** ***Il*** **-** ***1β*** **,** ***iNos*** **,** ***Bax*** **,** **and** ***Bcl2*** **.** Pretreatment of OUA can decrease the mRNA levels of pro-inflammatory cytokines and pro-apoptotic proteins. Representative semiquantitative RT- PCR photographs and densitometric analysis (arbitrary units, AU) of the specific bands. mRNA levels are presented as ratios of the target gene to *Gapdh* expression. Data are presented as mean ± SEM from five individual experiments. One-way ANOVA followed by Newman-Keuls post-test. **(A)** *Il-1β* mRNA,*P < 0.05 versus control and OUA and **P < 0.01 versus control and OUA and OUA + LPS. **(B)** *iNos* mRNA, *P < 0.05 versus control and OUA + LPS. **(C)** *Bax/Bcl2* ratio,**P < 0.001 versus OUA, control and OUA + LPS and *P < 0.05 versus OUA.

*iNos* mRNA levels, an important indicator of inflammation, increased in the LPS group in comparison with the control group as expected. OUA pretreatment decreased the LPS induction of *iNos* mRNA activation, returning levels to that of controls. OUA alone had no impact on *iNos* mRNA level (Figure 2A). *Il*-*1β* mRNA also increased in the LPS-treated group, which OUA pretreatment attenuated. OUA itself had no impact on *Il*-*1β* mRNA level (Figure 2B).

We also analyzed changes induced by LPS in the presence or absence of OUA on the ratio between the pro-apoptotic protein, *Bax*, and the anti-apoptotic protein, *Bcl2*. LPS increased the hippocampal *Bax*/*Bcl2* ratio, which OUA pretreatment prevented, suggesting an anti-apoptotic effect of OUA against LPS. Together, these data suggest that OUA has anti-inflammatory and anti-apoptotic effects.

### Pretreatment with OUA can block LPS-induced NF-κB translocation

**Figure 3:**
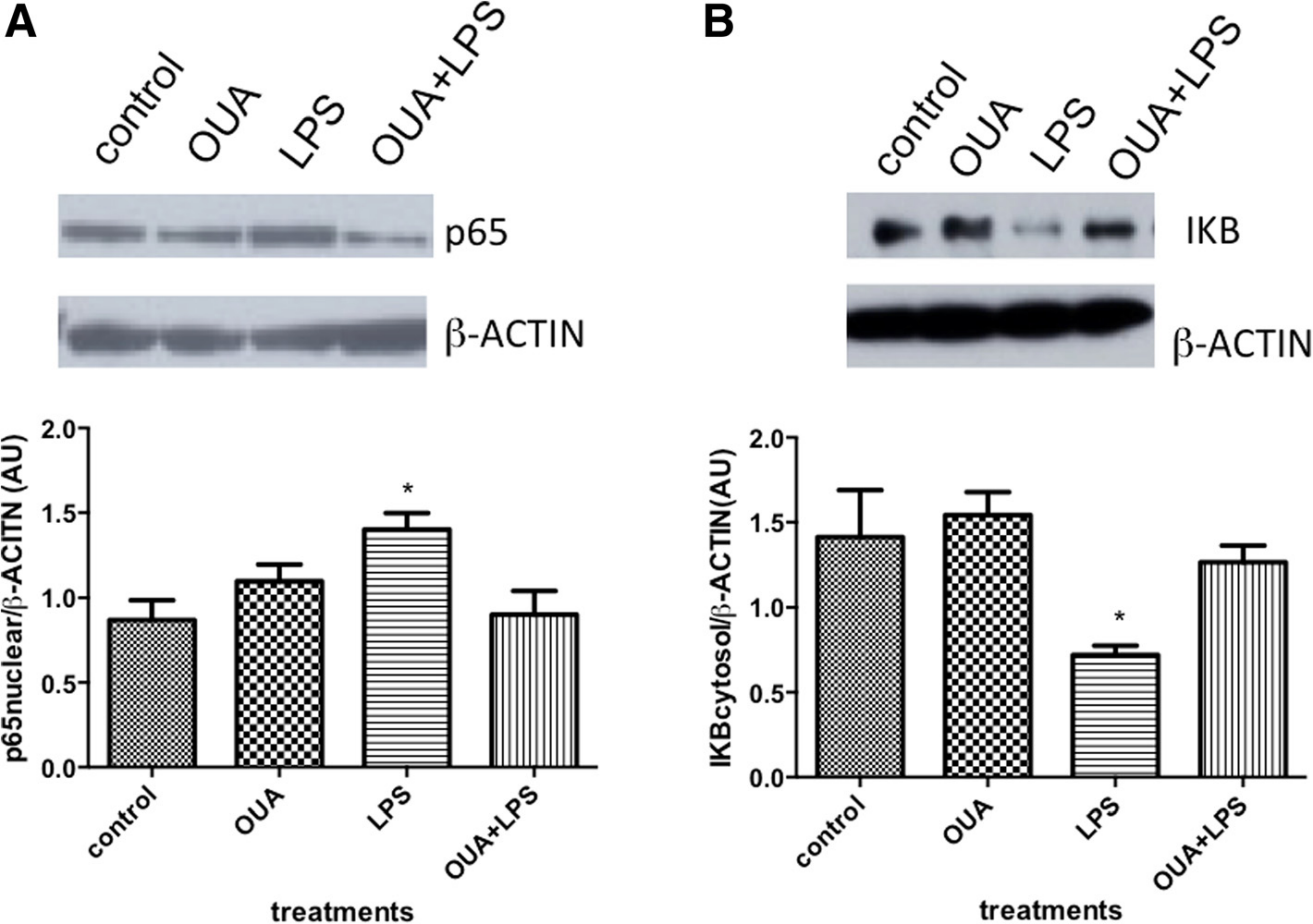
**Effects of pretreatment of ouabain (OUA)** **(1.8 μg/** **kg)** **on lipopolysaccharide (LPS)-induced** **(200 μg**/**kg** **) p65** **(RELA)** **subunit NF-** **κB nuclear translocation and IκB (IKB) degradation.** Pretreatment of OUA blocks the LPS-induced NF-κB activation. **(A)** Representative Western blotting autoradiographs and densitometric analysis (arbitrary units, AU) of p65 nuclear/β-ACTIN ratio, *P < 0.05 versus control and OUA + LPS. **(B)** Representative Western blotting autoradiographs and densitometric analysis (arbitrary units, AU) of IKB cytosolic/β-ACTIN ratio, *P < 0.05 versus control, OUA, OUA + LPS. Data are presented as mean ± SEM from five individual experiments. One-way ANOVA followed by Newman-Keuls post-test.

Western blotting assays showed that LPS (ip) administration increases both nuclear RELA (p65) translocation and cytosolic IKB degradation, which could lead to NF-κB activation in the rat hippocampus. Although OUA itself did not change either nuclear p65 translocation or cytosolic IKB, it did block LPS-induced RELA (p65) and IKB effects in the rat hippocampus (Figure 3A and B). The inhibition of LPS-induced NF-κB translocation by OUA likely underlies the OUA inhibition of LPS-induced *iNos*, *Il-1β* and increased *Bax/Bcl-2* ratio.

### Pretreatment with OUA can reverse the activation of astrocytes

**Figure 4:**
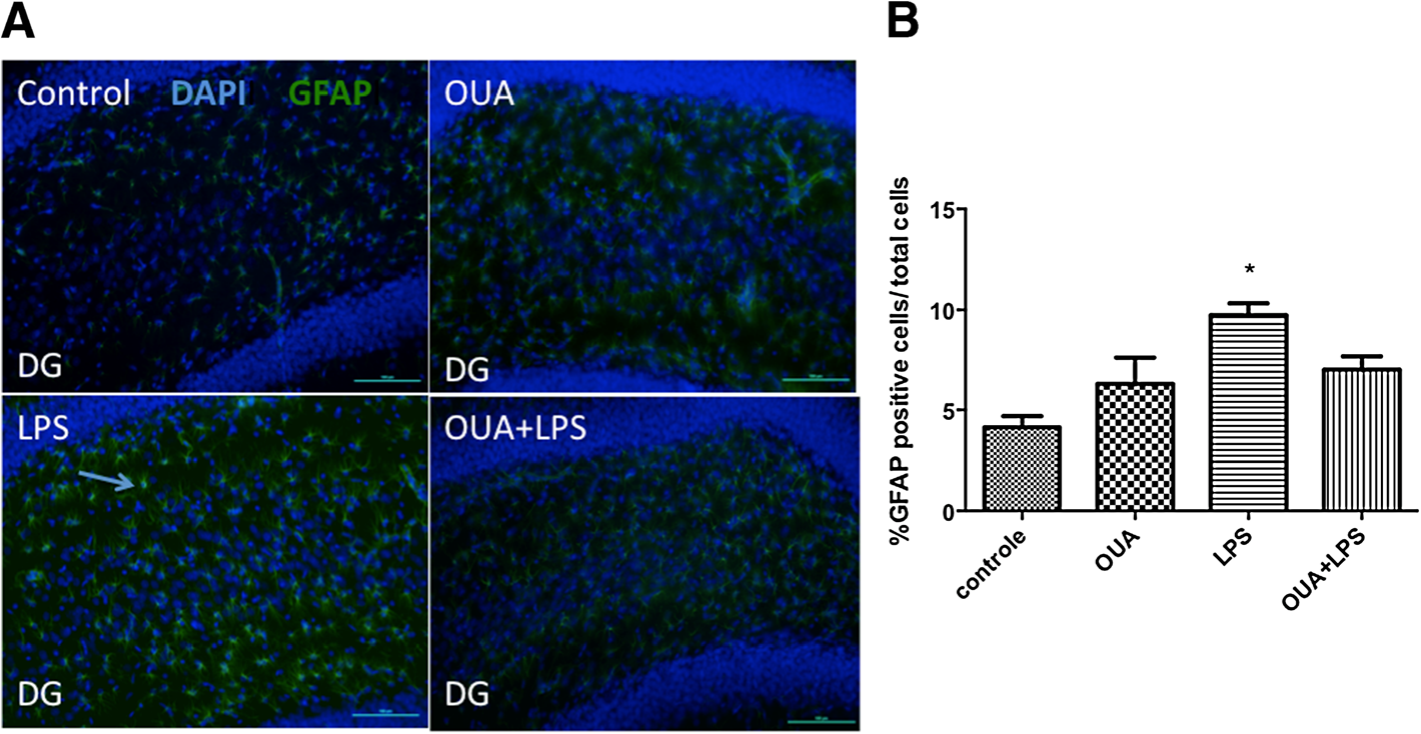
**The pretreatment of ouabain (OUA)** **(1.8 μg/** **kg)** **on lipopolysaccharide (LPS)-induced** **(200 μg** **/kg)** **astrocyte activation.** Astrocyte activity was measure with glial fibrillary acidic protein (GFAP) immunofluorescence in the dentate gyrus. **(A)** Representative photomicrographs showed that LPS increases astrocytic activation, with OUA pretreatment reducing this. The arrow represents an activated astrocyte. **(B)** Densitometric analysis of the % of GFAP-positive cells/total cells. *P < 0.05 versus control, OUA and OUA + LPS. Data are presented as mean ± SEM from five individual experiments. One-way ANOVA followed by Newman-Keuls post-test.

GFAP immunofluorescence of the dentate gyrus region of the hippocampus indicated that LPS induced astrocyte activation, which OUA pretreatment inhibits (Figure 4). Increased GFAP expression indicates astrocyte activation, showing that OUA pretreatment not only has an effect on pro-inflammatory cytokine levels, but also on the activation of pro-inflammatory cells in the dentate gyrus. Such data indicate OUA to be neuroprotective, at least in part by decreasing astrocyte activation.

### OUA does not reverse LPS-induced increase in serum corticosterone and TNF-α levels

**Figure 5:**
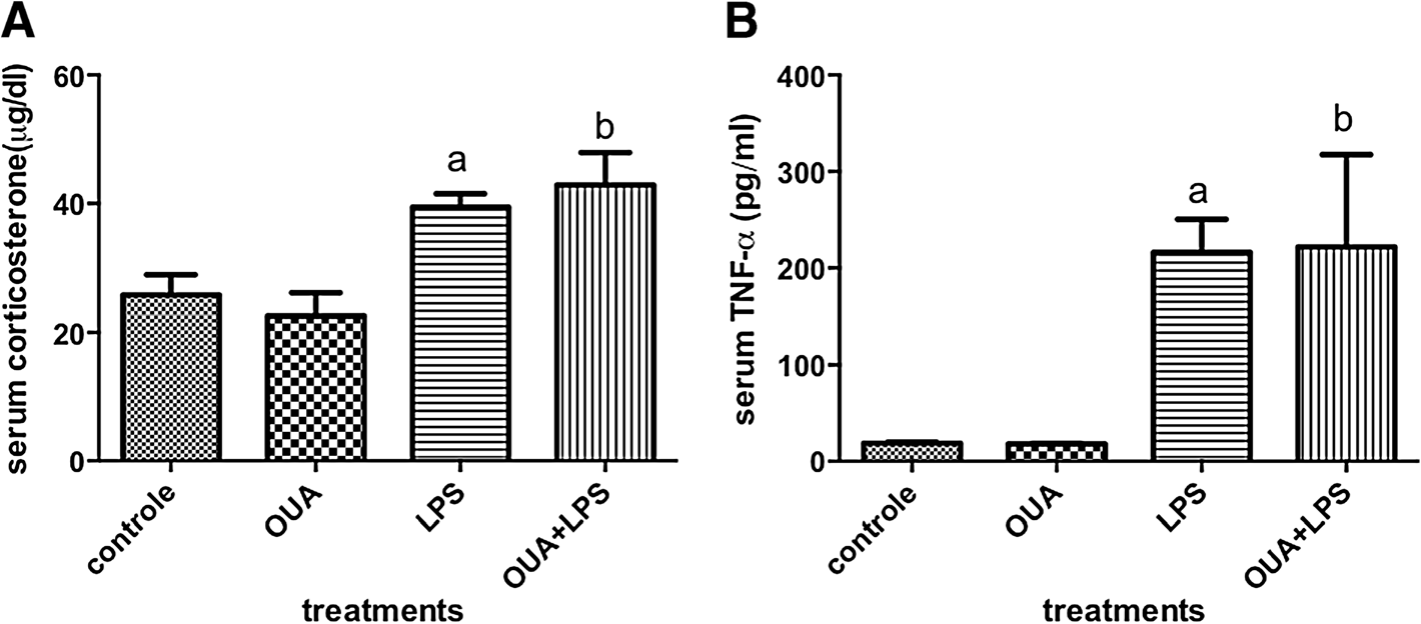
**Effects of ouabain (OUA) pretreatment** **(1.8 μg/** **kg)** **on lipopolysaccharide (LPS)-induced** **(200 μg/** **kg)** **serum corticosterone and TNF-** **α. (A)** The serum levels of corticosterone were elevated in LPS and OUA + LPS in comparison with control and OUA group, ^a^ *P* < 0.01 versus control and OUA and ^b^ *P* < 0.01 versus control and OUA. **(B)** The same pattern occurs in TNF-α serum levels, ^a^ *P* < 0.05 versus control and OUA and ^b^ *P* < 0.05 versus control and OUA. Data are presented as mean ± SEM from five individual experiments. One-way ANOVA followed by Newman-Keuls post-test.

Our data showed that LPS, but not OUA, increased serum levels of corticosterone and TNF-α (Figure 5A and B). In addition, OUA pretreatment was unable to block LPS-induced increases in serum corticosterone and TNF-α.

### OUA can modulate TNF-α and BDNF

**Figure 6:**
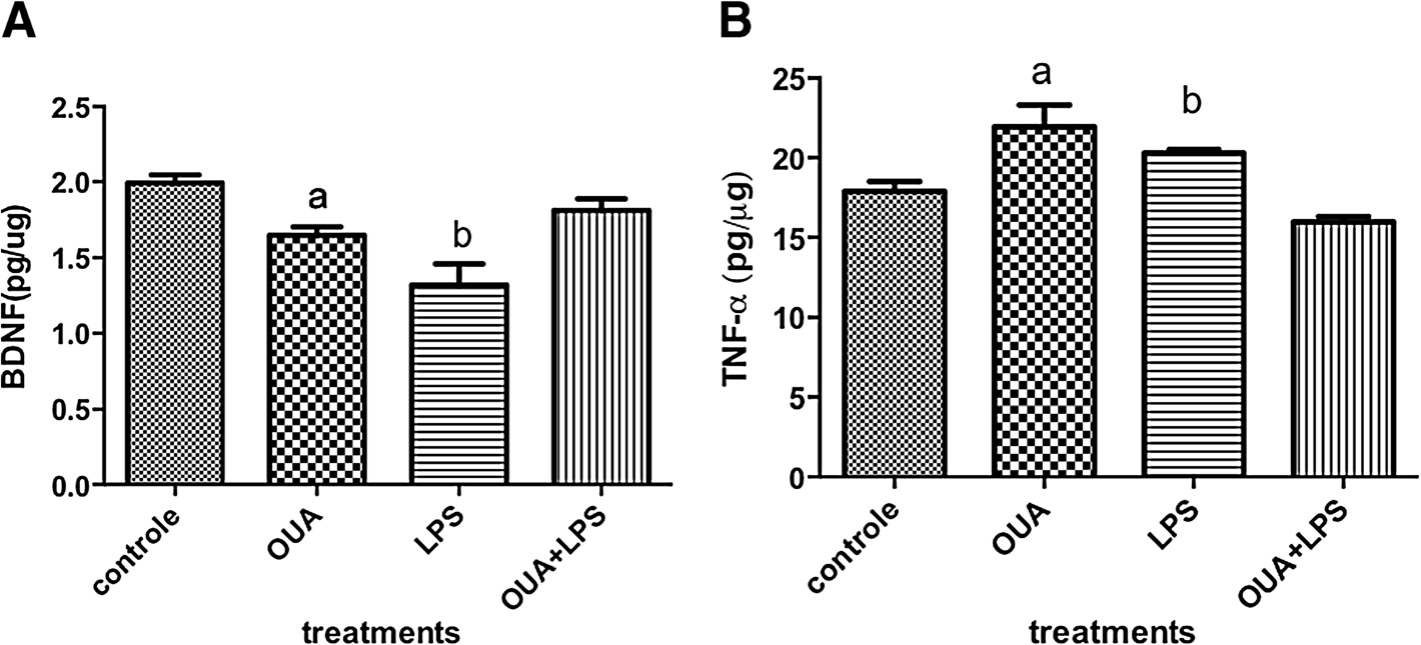
**Effects of ouabain (OUA) pretreatment** **(1.8 μg/** **kg)** **on lipopolysaccharide (LPS)-induced** **(200 μg/** **kg)** **regulation of hippocampal brain-derived neurotrophic factor (BDNF) and TNF**-**α levels. (A)** The levels of BDNF were decreased in LPS and OUA groups in comparison with control and OUA + LPS group, ^a^ *P* < 0.05 versus control and LPS and ^b^ *P* < 0.01 versus control and OUA + LPS and *P* < 0.05 versus OUA. **(B)** The levels of TNF-α in the hippocampus, ^a^ *P* < 0.01 versus control and OUA + LPS and ^b^ *P* < 0.05 versus control and OUA + LPS. Data are presented as mean ± SEM from five individual experiments. One-way ANOVA followed by Newman-Keuls test.

OUA or LPS alone significantly decreased BDNF in comparison to controls, although the decrease induced by LPS was significantly greater. However, OUA pretreatment reversed LPS effects, leading to BDNF levels at that of controls (Figure 6A). Likewise, LPS or OUA treatment alone increased the inflammatory cytokine, TNF-α, with OUA pretreatment preventing LPS-induced TNF-α increases versus control (Figure 6B).

This suggests protection of the rat hippocampus by OUA pretreatment against the effects of LPS, given that TNF-α is an important early pro-inflammatory cytokine, and BDNF is a significant neuronal protectant, as well as being a growth factor.

## Discussion

Neuroinflammation can be associated with neurological insults and neurodegenerative disorders. Neuroinflammation is characterized by increased pro-inflammatory cytokines, such as TNF-α and the expression on endothelial cells of adhesion molecules that increase leukocyte infiltration over the blood–brain barrier (BBB). Concurrently, astrocytes and microglia are activated [43].

LPS is an inflammation model that can activate NF-κB, partly mediated by glutamate acting at N-methyl-D-aspartate (NMDA) receptors, in turn increasing calcium-dependent activation of NOS and reactive oxygen species generation; the latter contributing to membrane peroxidation [36]. NF-κB activation induces the expression of many inflammatory cytokines and inflammatory factors that drive the innate immune response [29,44].

However, it is unpredictable as to whether circulating high concentrations of LPS increase BBB permeability, leading to the entry of LPS and other molecules into the brain parenchyma [36,37]. In the early stages of LPS infection, central areas not protected by the BBB are likely to contribute to the inflammatory process, including the actions of LPS at TLR4 in the pineal gland, which inhibits pineal melatonin production, in turn contributing to increased BBB permeability and immune system responsivity (reviewed in [45]).

TLR4 is also constitutively expressed in the leptomeninges, choroid plexus, subfornical organ, organum vasculosum of the lamina terminalis, median eminence, and area postrema. LPS effects in these non-BBB protected areas are likely to influence the inflammatory response in BBB protected areas, such as the hippocampus [46]. As such, LPS inflammatory effects may not be dependent on increased BBB permeability. The activation of inflammatory cytokines by LPS in this study confirms the results of previous studies from our laboratory, which showed that most of the transcriptional activity regulated by LPS administration is mediated by the p50/p65 dimer of NF-κB [36,38]. LPS intraperitoneally causes astrocyte activation in the dentate gyrus in aged rats, with this effect can be prolonged for many days [47]. Inflammation in the dentate gyrus can block neurogenesis in adult rats, which can be restored by an anti-inflammatory drug [48].

It is known that OUA can produce negative effects in a dose-dependent manner, but many studies have shown OUA to afford protection centrally and peripherally. In a serum deprivation model in rat embryonic kidney cell cultures, OUA prevents cell death by the activation of NF-κB [49]. It has also been shown that OUA can protect proximal tubular cells against Shiga toxin by activating the anti-apoptotic Bcl-xL [27].

Although OUA circulates in blood plasma of resting humans in the subnanomolar-to-nanomolar concentration range, non-inhibitory subnanomolar concentration of OUA might be linked to activation of a signal transducer pathway in rats since the Km for OUA inhibition of NKA is 10 to 100 μM for isolated rat vascular smooth muscle, whilst in humans it is 10 to 100 nM [50,51].

Thus, the OUA concentration (1.8 μg/kg, ip) used in the present study is likely linked to activation of the NKA signaling cascade, since in this study we used an OUA concentration that had no significant impact on blood pressure and it did not inhibit hippocampal NKA. As such, the results here are not dependent on NKA inhibition, but are dependent on changes in NKA-related signaling. Our data that showed ip LPS administration increased the mRNA levels of *iNos* and *Il*-*1β*, as well as the *Bax*/*Bcl2* ratio, whereas OUA alone had no impact on the mRNA levels of these factors. However, we observed a protective effect of OUA, given its decrease in the mRNA levels of *iNos* and *Il*-*1β*, as well as a decrease in the *Bax*/*Bcl2* ratio induced by LPS in the hippocampus.

This protection is likely to be afforded by OUA blocking LPS-induced nuclear translocation of NF-κB via the RELA (p65) subunit and by decreasing the degradation of cytosolic IKB, thereby maintaining IKB linked to NF-κB in the cytosol. The major OUA protective effect occurred in the adult neurogenesis-associated dentate gyrus [52].

Previous studies from our laboratory have reported that OUA can increase cytokine levels. Such variability in OUA effects may be due to a number of factors, including testing times and type of administration, as well as different tissue and cell models. In fact, we detected changes in cytokines gene expression 1 hour after injecting OUA directly in to the CA1 region of the hippocampus at a concentration of 10 nM [53], as well as in *in vitro* studies (OUA = 10 μM) in cerebellar primary culture cells [54].

TLR4 decreases neurogenesis and neuronal differentiation, with consequences for neuronal plasticity and memory [55]. BDNF increases neurogenesis, improves memory acquisition and has anti-apoptotic and anti-inflammatory effects, as well as protecting against hypoxia/ischemia-induced brain injury [56]. OUA pretreatment prevented the decrease in BDNF by LPS, although OUA alone also decreased BDNF. In fact, we cannot rule out that BDNF can also be modulated by other transcription factors that might be activated by OUA. OUA has both toxic [57,58] and protective actions against damage to cells [59,60], including neurons [26]. In the present study, OUA pretreatment reverted LPS-induced decreases in BDNF levels, suggesting a specific action of this compound against the inflammatory signaling response. OUA pretreatment also provided protection against LPS in the rat hippocampus by decreasing the LPS induction of the pro-inflammatory cytokine, TNF-α. OUA alone increased TNF-α, as we found previously [53,54]. This is surprising as another study showed OUA to block the activation of the TNF/NF-κB pathway by interfering with the interaction between TNF receptor 1 and TRADD in HeLa cell cultures [61]. Further work should clarify the temporal regulation of TNF-α and/or BDNF by OUA and also its perhaps differential effects in different cell types. In the periphery, our data showed that LPS, but not OUA, increased serum levels of corticosterone and TNF-α, with OUA pretreatment unable to block these changes. This could suggest that the OUA concentration used in the present study may be too low to reverse LPS peripheral activation, although it was high enough to protect against LPS effects in the hippocampus. This would imply that OUA protection could be mediated by its direct action in the brain. Alternatively, the peripheral effect of LPS may be stronger, with the OUA concentration used in the current study unable to reverse the peripheral effects of LPS.

In the peripheral system, mice treated with zymosan and carrageenan leads to paw edema, with OUA decreasing the inflammatory response in these models, suggesting that OUA has peripheral anti-inflammatory effects [62]. However, the OUA concentrations used were higher than in this current study, with the anti-inflammatory effects being mediated by different intracellular pathways, involving the inhibition of prostaglandin E2, bradykinin and mastocyte degranulation [62]. However, it should be noted that other mechanisms may be involved in the anti-inflammatory effects of steroid glycosides, including decreasing IL-6 [63] and inhibition of genes activated by TNF-α [64].

Recent studies have shown that low doses of OUA have anti-inflammatory properties in LPS-treated reactive astrocytes, by reducing IL-1β release. OUA also prevented the LPS-induced downregulation of NKA and restored the actin filaments in reactive astrocytes [65]. This is in contrast to the effects of OUA in LPS-treated microglia, where this compound had no effect on levels of microglial reactivity [66]. This could suggest that some of the differential effects of OUA on central processing are determined by the relative levels of microglial and astrocyte activation, including at different time points in the inflammatory process.

## Conclusions

Taken together, the present work showed that OUA pretreatment has anti-inflammatory and anti-apoptotic effects in the hippocampus challenged with LPS-induced inflammation. This effect is mediated by NF-κB activation, including in the neurogenesis-associated dentate gyrus. The ability of OUA to suppress the inflammatory process and maintain hippocampal BDNF levels in the face of inflammatory activity suggests that the NKA signaling cascade could be a new therapeutic target in neuroinflammation-associated disorders.

## Abbreviations

ANOVA: analysis of variance
ATP: adenosine triphosphate
BBB: blood–brain barrier
BDNF: brain-derived neurotrophic factor
bp: base pair
CAPOS: cerebellar ataxia, areflexia, pes cavus, optic atrophy and sensorineural hearing loss
CEEA: Ethical Committee for Animal Research
COBEA: Biomedical College of Animal Experimentation
CNS: central nervous system
DAPI: 4',6-diamidino-2-phenylindole
DTT: dithiothreitol
EDTA: ethylenediaminetetraacetic acid
ECL: electrochemiluminescence
EGFR: epidermal growth factor receptor
GAPDH: glyceraldehyde-3-phosphate dehydrogenase
GFAP: glial fibrillary acidic protein
IKB: NF-κB inhibitor protein
IKK: IκB kinase
IL-1β: interleukin 1 beta
iNOS: inducible nitric oxide synthase
ip: intraperitoneal
InsP3R: inositol trisphosphate receptor
Km: Michaelis-Menten equilibrium constant
LPS: lipopolysaccharide
MnSOD: manganese-superoxide dismutase
NF-κB: nuclear factor kappa B
NKA: Na,K-ATPase
NMDA: N-methyl-D-aspartate
OUA: ouabain
PBS: phosphate-buffered saline
PCR: polymerase chain reaction
PMSF: phenylmethylsulfonide fluoride
TLR4: toll-like receptor 4
TNF-α: tumor necrosis factor alpha

## References

[1] Schoner W (2000). Ouabain, a new steroid hormone of adrenal gland and hypothalamus. Exp Clin Endocrinol Diabetes.

[2] Kawamura A, Guo J, Itagaki Y, Bell C, Wang Y, Haupert GT, Magil S, Gallagher RT, Berova N, Nakanishi K (1999). On the structure of endogenous ouabain. Proc Natl Acad Sci U S A.

[3] Albers RW (1999). Cell Membrane Structures and Functions.

[4] Fambrough DM (1988). The sodium pump becomes a family. Trends Neurosci.

[5] Levenson R (1994). Isoforms of the Na, K-ATPase: family members in search of function. Rev Physiol Biochem Pharmacol.

[6] Lingrel JB, Kuntzweiler T (1994). Na+, K(+)-ATPase. J Biol Chem.

[7] Sweadner KJ: **Isozymes of the Na**^**+**^**/K**^**+**^**-ATPase.** *Biochim Biophys Acta* 1989, **988:**185–220. 10.1016/0304-4157(89)90019-1 2541792

[8] Sweadner KJ: **Overview: subunit diversity in the Na**^**+**^**/K**^**+**^**-ATPase.** *Soc Gen Physiol Ser* 1991, **46:**63–76. 1653994

[9] Minor N, Sha Q, Nichols C, Mercer R (1998). The gamma subunit of the Na, K-ATPase induces cation channel activity. Proc Natl Acad Sci U S A.

[10] Beltowski J, Wójcicka G (2002). Regulation of renal tubular sodium transport by cardiac natriuretic peptides: two decades of research. Med Sci Monit.

[11] Peng L, Arystarkhova E, Sweadner KJ (1998). Plasticity of Na, K-ATPase isoform expression in cultures of flat astrocytes: species differences in gene expression. Glia.

[12] De Fusco M, Marconi R, Silvestri L, Atorino L, Rampoldi L, Morgante L, Ballabio A, Aridon P, Casari G (2003). Haploinsufficiency of ATP1A2 encoding the Na+/K+ pump alpha2 subunit associated with familial hemiplegic migraine type 2. Nat Genet.

[13] Moseley AE, Williams MT, Schaefer TL, Bohanan CS, Neumann JC, Behbehani MM, Vorhees CV, Lingrel JB (2007). Deficiency in Na, K-ATPase alpha isoform genes alters spatial learning, motor activity, and anxiety in mice. J Neurosci.

[14] Heinzen EL, Swoboda KJ, Hitomi Y, Gurrieri F, Nicole S, de Vries B, Tiziano FD, Fontaine B, Walley NM, Heavin S, Panagiotakaki E, Fiori S, Abiusi E, Di Pietro L, Sweney MT, Newcomb TM, Viollet L, Huff C, Jorde LB, Reyna SP, Murphy KJ, Shianna KV, Gumbs CE, Little L, Silver K, Ptáček LJ, Haan J, European Alternating Hemiplegia of Childhood (AHC) Genetics Consortium, Biobanca e Registro Clinico per l'Emiplegia Alternante (I.B.AHC) Consortium, European Network for Research on Alternating Hemiplegia (ENRAH) for Small and Medium-sized Enterpriese (SMEs) Consortium (2012). *De novo* mutations in ATP1A3 cause alternating hemiplegia of childhood. Nat Genet.

[15] Demos MK, van Karnebeek CD, Ross CJ, Adam S, Shen Y, Zhan SH, Shyr C, Horvath G, Suri M, Fryer A, Jones SJ, Friedman JM, FORGE Canada Consortium (2014). A novel recurrent mutation in ATP1A3 causes CAPOS syndrome. Orphanet J Rare Dis.

[16] Goldstein I, Lerer E, Laiba E, Mallet J, Mujaheed M, Laurent C, Rosen H, Ebstein RP, Lichtstein D (2009). Association between sodium- and potassium-activated adenosine triphosphatase alpha isoforms and bipolar disorders. Biol Psychiatry.

[17] Zhang LN, Sun YJ, Pan S, Li JX, Qu YE, Li Y, Wang YL, Gao ZB (2013). Na(+)-K(+)-ATPase, a potent neuroprotective modulator against Alzheimer disease. Fundam Clin Pharmacol.

[18] Aizman O, Aperia A (2003). Na, K-ATPase as a signal transducer. Ann N Y Acad Sci.

[19] Haas M, Wang H, Tian J, Xie Z (2002). Src-mediated inter-receptor cross-talk between the Na+/K + −ATPase and the epidermal growth factor receptor relays the signal from ouabain to mitogen-activated protein kinases. J Biol Chem.

[20] Murrell JR, Randall JD, Rosoff J, Zhao JL, Jensen RV, Gullans SR, Haupert GT (2005). Endogenous ouabain: upregulation of steroidogenic genes in hypertensive hypothalamus but not adrenal. Circulation.

[21] El-Masri MA, Clark BJ, Qazzaz HM, Valdes R (2002). Human adrenal cells in culture produce both ouabain-like and dihydroouabain-like factors. Clin Chem.

[22] Liu X, Spicarova Z, Rydholm S, Li J, Brismar H, Aperia A (2008). Ankyrin B modulates the function of Na, K-ATPase/inositol 1,4,5-trisphosphate receptor signaling microdomain. J Biol Chem.

[23] Liu XL, Miyakawa A, Aperia A, Krieger P (2007). Na, K-ATPase generates calcium oscillations in hippocampal astrocytes. Neuroreport.

[24] Xie Z, Askari A (2002). Na(+)/K(+)-ATPase as a signal transducer. Eur J Biochem.

[25] Xiao AY, Wei L, Xia S, Rothman S, Yu SP (2002). Ionic mechanism of ouabain-induced concurrent apoptosis and necrosis in individual cultured cortical neurons. J Neurosci.

[26] Golden WC, Martin LJ (2006). Low-dose ouabain protects against excitotoxic apoptosis and up-regulates nuclear BLC-2 *in vivo*. Neuroscience.

[27] Burlaka I, Liu XL, Rebetz J, Arvidsson I, Yang L, Brismar H, Karpman D, Aperia A (2013). Ouabain protects against Shiga toxin-triggered apoptosis by reversing the imbalance between Bax and Bcl-xL. J Am Soc Nephrol.

[28] Sen R, Baltimore D (1986). Multiple nuclear factors interact with the immunoglobulin enhancer sequences. Cell.

[29] Ghosh S, May MJ, Kopp EB (1998). NF-kappa B and Rel proteins: evolutionarily conserved mediators of immune responses. Annu Rev Immunol.

[30] Baeuerle PA, Baltimore D (1996). NF-kappa B: ten years after. Cell.

[31] Zandi E, Rothwarf DM, Delhase M, Hayakawa M, Karin M (1997). The IkappaB kinase complex (IKK) contains two kinase subunits, IKKalpha and IKKbeta, necessary for IkappaB phosphorylation and NF-kappaB activation. Cell.

[32] Mattson MP (2008). Glutamate and neurotrophic factors in neuronal plasticity and disease. Ann N Y Acad Sci.

[33] Camandola S, Mattson MP (2007). NF-kappa B as a therapeutic target in neurodegenerative diseases. Expert Opin Ther Targets.

[34] Glaros TG, Chang S, Gilliam EA, Maitra U, Deng H, Li L (2013). Causes and consequences of low grade endotoxemia and inflammatory diseases. Front Biosci (Schol Ed).

[35] Kawamoto EM, Scavone C, Mattson MP, Camandola S (2013). Curcumin requires tumor necrosis factor alpha signaling to alleviate cognitive impairment elicited by lipopolysaccharide. Neurosignals.

[36] Glezer I, Munhoz CD, Kawamoto EM, Marcourakis T, Avellar MC, Scavone C (2003). MK-801 and 7-Ni attenuate the activation of brain NF-kappa B induced by LPS. Neuropharmacology.

[37] Laflamme N, Rivest S (1999). Effects of systemic immunogenic insults and circulating proinflammatory cytokines on the transcription of the inhibitory factor kappaB alpha within specific cellular populations of the rat brain. J Neurochem.

[38] Munhoz CD, Lepsch LB, Kawamoto EM, Malta MB, Lima Lde S, Avellar MC, Sapolsky RM, Scavone C (2006). Chronic unpredictable stress exacerbates lipopolysaccharide-induced activation of nuclear factor-kappaB in the frontal cortex and hippocampus via glucocorticoid secretion. J Neurosci.

[39] Rong Y, Baudry M (1996). Seizure activity results in a rapid induction of nuclear factor-kappa B in adult but not juvenile rat limbic structures. J Neurochem..

[40] Salinovich O, Montelaro R (1986). Reversible staining and peptide mapping of protein transferred to nitrocellulose after separation by SDS-PAGE. Anal Biochem.

[41] Esmann M (1988). ATPase and phosphatase activity of Na+, K + −ATPase: molar and specific activity, protein determination. Methods Enzymol.

[42] Bradford MM (1976). A rapid and sensitive method for the quantitation of microgram quantities of protein utilizing the principle of protein-dye binding. Anal Biochem.

[43] Gonzales-Scarano F, Baltuch G (1999). Microglia as mediators of inflammatory and degenerative diseases. Annu Rev Neurosci.

[44] Heese K, Fiebich B, Bauer J, Otten U (1998). NF-kappaB modulates lipopolysaccharide-induced microglial nerve growth factor expression. Glia.

[45] Markus RP, Cecon E, Pires-Lapa MA (2013). Immune-pineal axis: nuclear factor κB (NF-kB) mediates the shift in the melatonin source from pinealocytes to immune competent cells. Int J Mol Sci.

[46] Laflamme N, Soucy G, Rivest S (2001). Circulating cell wall components derived from Gram-negative, not Gram-positive, bacteria cause a profound induction of the gene-encoding Toll-like receptor 2 in the CNS. J Neurochem.

[47] Fu H, Yang T, Xiao W, Fan L, Wu Y, Terrando N, Wang T (2014). Prolonged neuroinflammation after lipopolysaccharide exposure in aged rats. Plos One.

[48] Monje M, Toda H, Palmer T (2003). Inflammatory blockade restores adult hippocampal neurogenesis. Science.

[49] Li J, Khodus GR, Kruusmagi M, Kamali-Zare P, Liu XL, Eklof AC, Zelenin S, Brismar H, Aperia A (2010). Ouabain protects against adverse developmental programming of the kidney. Nat Commun.

[50] Orlov SN, Taurin S, Hamet P (2002). The alpha1-Na/K pump does not mediate the involvement of ouabain in the development of hypertension in rats. Blood Press.

[51] Schoner W, Scheiner-Bobis G (2007). Endogenous and exogenous cardiac glycosides: their roles in hypertension, salt metabolism, and cell growth. Am J Physiol Cell Physiol.

[52] Cameron H, McKay R (2001). Adult neurogenesis produces a large pool of new granule cells in the dentate gyrus. J Comp Neurol.

[53] Kawamoto EM, Lima LS, Munhoz CD, Yshii LM, Kinoshita PF, Amara FG, Pestana RR, Orellana AM, Cipolla-Neto J, Britto LR, Avellar MC, Rossoni LV, Scavone C (2012). Influence of N-methyl-D-aspartate receptors on ouabain activation of nuclear factor-kappaB in the rat hippocampus. J Neurosci Res.

[54] de Sa LL, Kawamoto EM, Munhoz CD, Kinoshita PF, Orellana AM, Curi R, Rossoni LV, Avellar MC, Scavone C (2013). Ouabain activates NFkappaB through an NMDA signaling pathway in cultured cerebellar cells. Neuropharmacology.

[55] Rolls A, Shechter R, London A, Ziv Y, Ronen A, Levy R, Schwartz M (2007). Toll-like receptors modulate adult hippocampal neurogenesis. Nat Cell Biol.

[56] Chen A, Xiong L, Tong Y, Mao M (2013). The neuroprotective roles of BDNF in hypoxic ischemic brain injury. Biomed Rep.

[57] Valente RC, Capella LS, Monteiro RQ, Rumjanek VM, Lopes AG, Capella MA (2003). Mechanisms of ouabain toxicity. FASEB J.

[58] Orlov SN, Thorin-Trescases N, Pchejetski D, Taurin S, Farhat N, Tremblay J, Thorin E, Hamet P (2004). Na/K pump and endothelial cell survival: [Na ]i/[K ]i-independent necrosis triggered by ouabain, and protection against apoptosis mediated by elevation of [Na]i. Pflugers Arch.

[59] Li J, Zelenin S, Aperia A, Aizman O (2006). Low doses of ouabain protect from serum deprivation-triggered apoptosis and stimulate kidney cell proliferation via activation of NF-kappaB. J Am Soc Nephrol.

[60] Pasdois P, Quinlan CL, Rissa A, Tariosse L, Vinassa B, Costa AD, Pierre SV, Dos Santos P, Garlid KD (2007). Ouabain protects rat hearts against ischemia-reperfusion injury via pathway involving src kinase, mitoKATP, and ROS. Am J Physiol Heart Circ Physiol.

[61] Yang Q, Huang W, Jozwik C, Lin Y, Glasman M, Caohuy H, Srivastava M, Esposito D, Gillette W, Hartley J, Pollard HB (2005). Cardiac glycosides inhibit TNF-alpha/NF-kappaB signaling by blocking recruitment of TNF receptor-associated death domain to the TNF receptor. Proc Natl Acad Sci U S A.

[62] de Vasconcelos DI, Leite JA, Carneiro LT, Piuvezam MR, de Lima MR, de Morais LC, Rumjanek VM, Rodrigues-Mascarenhas S (2011). Anti-inflammatory and antinociceptive activity of ouabain in mice. Mediators Inflamm.

[63] Matsumori A, Ono K, Nishio R, Igata H, Shioi T, Matsui S, Furukawa Y, Iwasaki A, Nose Y, Sasayama S (1997). Modulation of cytokine production and protection against lethal endotoxemia by the cardiac glycoside ouabain. Circulation.

[64] Ye J, Chen S, Maniatis T (2011). Cardiac glycosides are potent inhibitors of interferon-beta gene expression. Nat Chem Biol.

[65] Forshammar J, Block L, Lundborg C, Biber B, Hansson E (2011). Naloxone and ouabain in ultralow concentrations restore Na+/K + −ATPase and cytoskeleton in lipopolysaccharide-treated astrocytes. J Biol Chem.

[66] Forshammar J, Jörneberg P, Björklund U, Westerlund A, Lundborg C, Biber B, Hansson E (2013). Anti-inflammatory substances can influence some glial cell types but not others. Brain Res.

